# miR-29a Is Downregulated in Progenies Derived from Chronically Stressed Males

**DOI:** 10.3390/ijms241814107

**Published:** 2023-09-14

**Authors:** Marta F. Riesco, David G. Valcarce, Alba Sellés-Egea, Anna Esteve-Codina, María Paz Herráez, Vanesa Robles

**Affiliations:** 1 INDEGSAL, Cell Biology Area, Molecular Biology Department, Universidad de León, Campus de Vegazana s/n, 24071 León, Spain; 2 CNAG-CRG, Centre for Genomic Regulation, Barcelona Institute of Science and Technology (BIST), 08028 Barcelona, Spain; 3 Department of Medicine and Health Sciences, Universitat Pompeu Fabra (UPF), 08002 Barcelona, Spain

**Keywords:** chronic stress, offspring, RNA-seq, small-RNAs, behavior, hatching rates, survival, malformations

## Abstract

Recent research has provided compelling evidence demonstrating that paternal exposure to different stressors can influence their offspring’s phenotypes. We hypothesized that paternal stress can negatively impact the progeny, altering different miRs and triggering different physiological alterations that could compromise offspring development. To investigate this, we exposed zebrafish male siblings to a chronic stress protocol for 21 days. We performed RNA-sequencing (RNA-seq) analyses to identify differentially expressed small noncoding RNAs in 7-day postfertilization (dpf) larvae derived from paternally stressed males crossed with control females compared with the control progeny. We found a single miRNA differentially expressed—miR-29a—which was validated in larva and was also tested in the sperm, testicles, and brain of the stressed progenitors. We observed a vertical transmission of chronic stress to the unexposed larvae, reporting novel consequences of paternally inherited chronic stress at a molecular level. The deregulation of mi-R29a in those larvae could affect relevant biological processes affecting development, morphogenesis, or neurogenesis, among others. Additionally, these disruptions were associated with reduced rates of survival and hatching in the affected offspring.

## 1. Introduction

Chronic stress (CS) is one of the main concerns of modern society, creating ongoing scientific debate. The concept of stress in a biological context can be defined as a physiological cascade of events that occurs when an organism tries to re-establish homeostasis in the presence of an adverse stimulus [1,2,3]. Repeated or prolonged exposure to stress might compromise such physiological adaptive response, affecting several biological processes—including reproductive capacity—and decreasing gamete quality in human populations [4,5,6].

In recent years, significant progress has been made in identifying the main molecular mechanisms through which stressful conditions can trigger or aggravate reproductive dysfunctions. The consequences of stress exposure can reach not only the individuals exposed but also their offspring [7]. Traditionally, studies in different species—from fish to humans—have focused on the impact of maternal exposure on the offspring phenotypes, ignoring the possibility of male contribution [8,9]. However, new evidence has revealed that paternal exposure to diverse environmental situations—including stress conditions—can condition the offspring phenotypes [10,11,12]. Recent research has proven a paternal transmission of stress phenotypes via sperm [13]. In this phenotype inheritance, different molecules—such as mRNAs and miRNAs (miRs)—can act as one of the vehicles of progeny transmission [14,15,16,17,18]. In a previous study published by our group, we found that the resulting progenies of stressed progenitors presented molecular alterations related to translation initiation, DNA repair, cell cycle control, and response to stress, which can potentially compromise offspring development [13].

miRNAs are considered important regulatory elements for transcriptional control during early embryogenesis [19]. Furthermore, some sperm miRNAs have been recently identified as molecular biomarkers of reproductive performance, controlling different processes such as spermatogenesis and playing important roles in the control of male fertility [19,20,21,22,23]. Sperm miRs have been increasingly recognized as responsible for transmitting paternal lifetime experiences—including stress exposure—to offspring, evading embryonic reprogramming [11,24]. Reduced levels of certain miRs in murine sperm and men when exposed to early life stress can contribute to the transmission of stressed phenotypes across generations [11]. These small ncRNAs are highly conserved among animal species—including humans—suggesting that these may act as a regulator of gene expression across different animal species [25,26].

For this reason, we use zebrafish (*Danio rerio*) in the present study as this represents a suitable model species in human research and the aquaculture field. Abundant literature has described the role of zebrafish in fish aquaculture research, using this model to study coping styles, immunology, toxicology, disease, nutrition, reproduction, and stress [27,28,29,30,31]. Regarding human research, large similarities have been identified with humans in two zebrafish physiological master axes: hypothalamus–pituitary–gonad (HPG) and hypothalamus–pituitary–interrenal (HPI), which control, respectively, the reproductive performance and the stress response [32]. These similarities make zebrafish a highly suitable species to study stress responses and their transmission to offspring and extrapolate the obtained results to different commercial fish species or even to humans [27,33].

**Figure 1:**
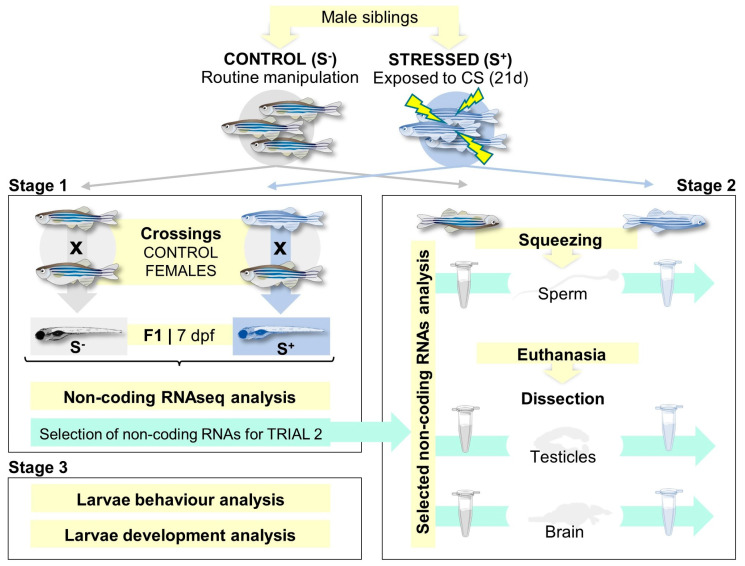
Experimental design. Zebrafish male siblings were exposed to a chronic stress (CS) protocol for 21 days. The three stages of the experiment are Stage 1, aimed to find differentially expressed noncoding RNAs in 7-day postfertilization (dpf) larvae derived from paternally chronically stressed males crossed with control females compared with control progenies; Stage 2, focused on evaluating the expression of the selected ncRNA on different paternal tissues (sperm, testicles, and brain); Stage 3, larvae behavioral and developmental analyses.

We hypothesize that paternal stress effects can impact the progeny, altering different miRs and triggering different physiological alterations that could compromise offspring development (Figure 1). We aim to shed light on the stress effect capacity for vertical transmission under a molecular prism, considering miRs as masterpieces of these molecular and phenotypical changes.

## 2. Results

### 2.1. RNAseq Analysis

#### 2.1.1. Differentially Expressed ncRNAs

**Figure 2:**
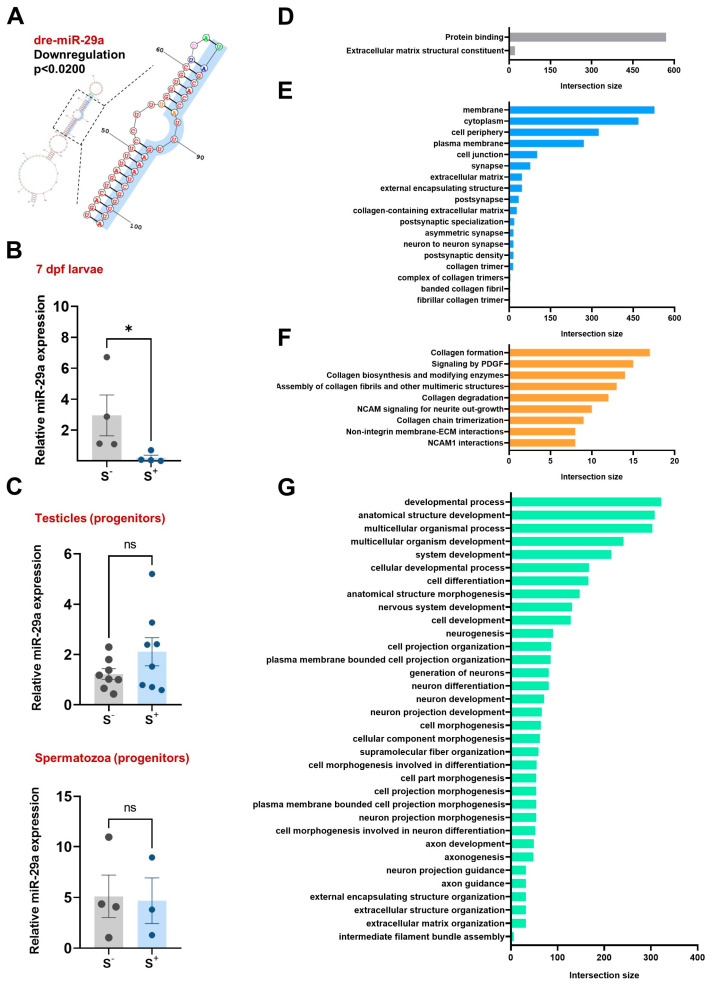
RNA-seq analyses focused on small RNAs populations in S^−^ and S^+^ 7 dpf larvae revealed (**A**) miR-29a as the unique differentially expressed small RNA. (**B**) validation of miR-29a downregulation with qPCR experiments. (**C**) miR-29a levels in the testicles (*n* = 8) and spermatozoa (*n* = 4 pools) samples from the chronically stressed male progenitors. Top entries reported by g:Profiler analysis of the miR-29a targets included in the TargetFishScan database for (**D**) molecular function, (**E**) cellular component, (**F**) biological pathways (Reactome database), and (**G**) biological processes. S^−^: larvae resulting from crossings involving control progenitors. S^+^: larvae from crossings involving control females and chronically stressed males. Data in (**B**,**C**) are presented as mean ± SEM (* *p* < 0.0500; ns: not statistically significant differences).

The RNA-seq analysis exclusively reported one noncoding RNA molecule differentially expressed between the two experimental groups: miR-29a. This microRNA exhibited a downregulation (*p* < 0.0200) in the S^+^ larvae group (Figure 2A).

#### 2.1.2. RNAseq Validation

The qPCR experiments conducted to validate the results obtained from RNAseq confirmed the downregulation (*p* = 0.0286) of miR-29a in the 7-day postfertilization (dpf) progeny derived from males exposed to CS (Figure 2B).

### 2.2. miR-29a Levels in Reproductive Cells and Tissues

Having established and confirmed the downregulation of miR-29a in larvae derived from chronically stressed parents, we aimed to assess whether the expression of this regulatory molecule was altered also in testicular and sperm samples. The qPCR results (Figure 2C) revealed similar miR-29a levels in samples obtained from control and stressed individuals for both testicular samples (*p* = 0.1717) and semen pools (*p* = 0.8953).

### 2.3. Exploration of miR-29a Targets

TargetScanFish reported a list of 2208 potentially miR-29a targeted mRNAs. The complete analysis of this list using g:Profiler is shown in Figure 2D–G.

In terms of cellular function, miR-29a targets are related to protein binding and extracellular matrix structural components (Figure 2D). Regarding cellular component analysis (Figure 2E), miR-29a targets exhibited significant intersections with membrane (GO:0016020), cytoplasm (GO:0005737), and cell periphery (GO:0071944). Notably, entries were reported related to extracellular matrix (extracellular matrix (GO:0031012); external encapsulating structure (GO:0030312) and collagen biosynthesis (collagen trimers (GO:0005581); collagen-containing extracellular matrix (GO:0062023); complex of collagen trimers (GO:0098644); fibrillar collagen trimer (GO:0005583); banded collagen fibril (GO:0098643), along with others associated with synapse (postsynaptic density (GO:0014069); neuron to neuron synapse (GO:0098984); asymmetric synapse (GO:0032279); postsynaptic specialization (GO:0099572); synapse (GO:0045202). An examination of biological pathways using Reactome unveiled a significant association with collagen biosynthesis, as five out of the nine statistically significant entries were related to collagen (Figure 2F). The analysis of biological processes (Figure 2G) determined a significant intersection including developmental process (GO:0032502), anatomical structure development (GO:0048856), multicellular organismal process (GO:0032501), multicellular organism development (GO:0007275), and system development (GO:0048731). Additional entries were associated with neural processes and morphogenesis.

### 2.4. Expression of miR-29a Targets in Larvae

**Figure 3:**
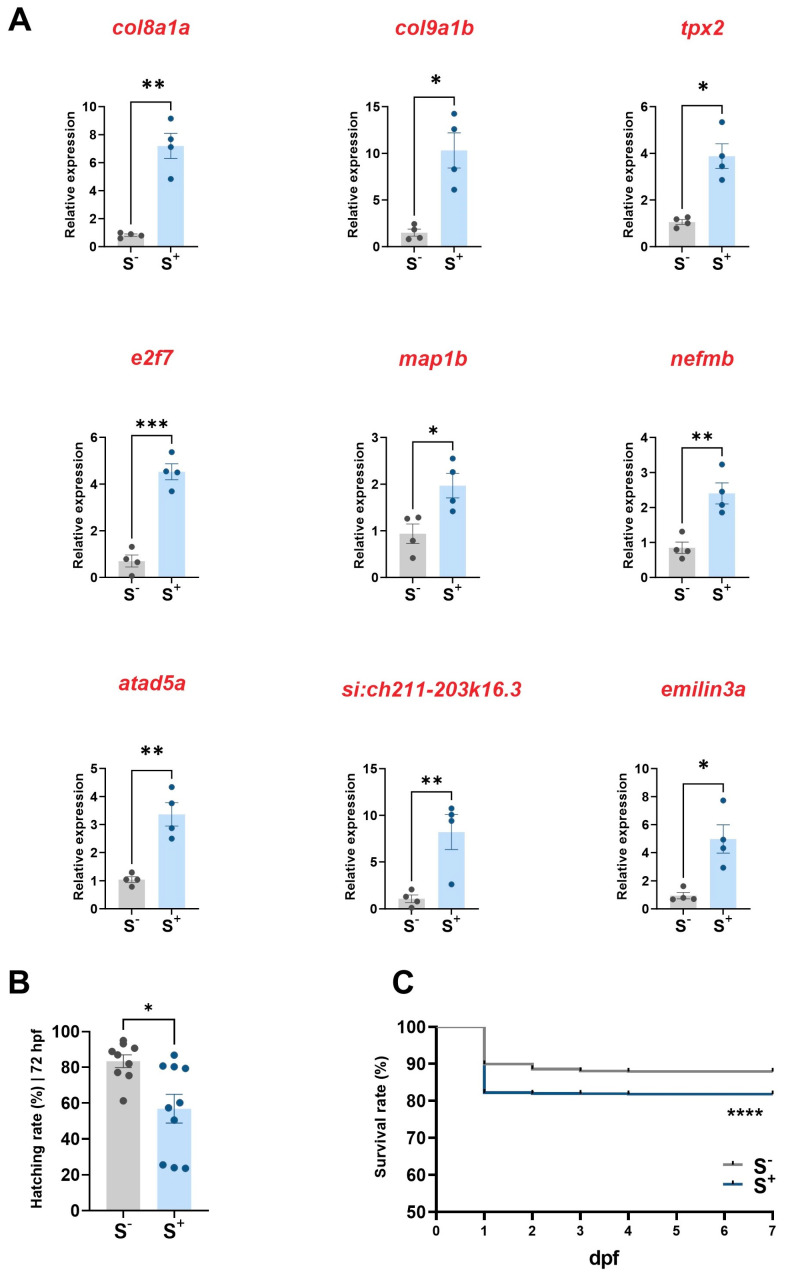
(**A**) relative gene expression (normalized with *actb2*) of nine targets of miR-29a in 7 dpf larvae (*n* = 4). (**B**) hatching rate at 72 h postfertilization (hpf; *n* = 9–10). (**C**) Kaplan–Meier survival curves (1–7 dpf) for the two experimental groups. S^−^: larvae resulting from crossings involving control progenitors. S^+^: larvae from crossings involving control females and chronically stressed males. Data are presented as mean ± SEM (* *p* < 0.0500; ** *p* < 0.0100; *** *p* < 0.0010; and **** *p* < 0.00010).

We obtained robust results with the evaluation of all selected miR-29a target genes using qPCR, indicating a widespread upregulation of these genes in the S^+^ experimental larvae group (Figure 3A). All nine genes exhibited statistically significant differences in their gene expression values: *col8a1a* (*p* = 0.0054), *col9a1b* (*p* = 0.0163), *tpx2* (*p* = 0.0112), *e2f7* (*p* = 0.0001), *map1b* (*p* = 0.0219), *nefmb* (*p* = 0.0040), *atad5a* (*p* = 0.0093), *si:ch211-203k16.3* (*p* = 0.0291), and *emilin3a* (*p* = 0.0286).

### 2.5. Progenies Development

#### 2.5.1. Hatching Rate

Comparison of hatching percentages at 72 h postfertilization (hpf) demonstrated statistically significant differences (*p* = 0.0246; Figure 3B). Specifically, larvae derived from chronically stressed male progenitors exhibited a lower hatching rate (56.80 ± 7.980%) compared with that of the progenies derived from undisturbed progenitors (83.32 ± 3.577%).

#### 2.5.2. Survival

The Kaplan–Meier survival curve comparison, assessed using the log-rank Mantel–Cox test, revealed statistically significant differences (*p* < 0.0001) between the progenies originating from the S^−^ and S^+^ larvae (Figure 3C). In the control group, the survival rate was approximately 87.90%, while the larvae derived from stressed conditions exhibited a mean survival rate of 81.78% at the end of the trial.

### 2.6. Brain and Behavioral Analyses

#### 2.6.1. miR-29a Levels in Parental Brain Samples

**Figure 4:**
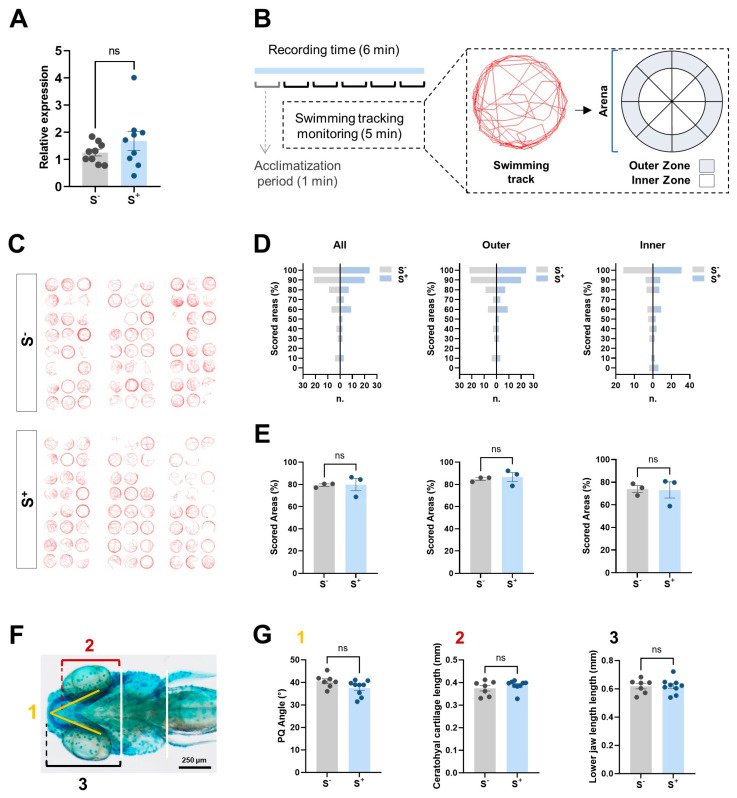
Brain and behavioral analyses. (**A**) relative miR-29a expression in paternal brains (*n* = 9). (**B**) diagram representing the novel tank test (NTT) used in the experiment. Example of a track reported by Tracker software 6.0.8 version (red line). Virtual grid (16 zones) used for movement quantification in terms of zone exploration. (**C**) evaluated tracks from both groups. (**D**) histograms showing scored areas in both groups studying the total areas (all), perimetral zones (outer), and central zones (inner). (**E**) mean values for the scoring areas for the biological replicates (*n* = 3). (**F**) diagram showing the three measurements evaluated in the cranioencephalic region of the larvae: (1) Meckel’s-palatoquadrate (M–PQ) angle, (2) ceratohyal cartilage length, and (3) lower jaw length. (**G**) comparisons for M–PQ angle, ceratohyal cartilage length (mm), lower jaw length (mm). S^−^: larvae resulting from crossings involving control progenitors. S^+^: larvae from crossings involving control females and chronically stressed males. Data are presented as mean ± SEM (ns: not statistically significant differences).

Given the impact of miR-29a targets on synapse-related pathways, we decided to explore the expression of this molecule in adult brains. qPCR analysis revealed no statistically significant differences (*p* = 0.2718) in the levels of miR-29a between the unstressed and the stressed parents (Figure 4A).

#### 2.6.2. Larvae and Adult Behavior Analysis

**Figure A1:**
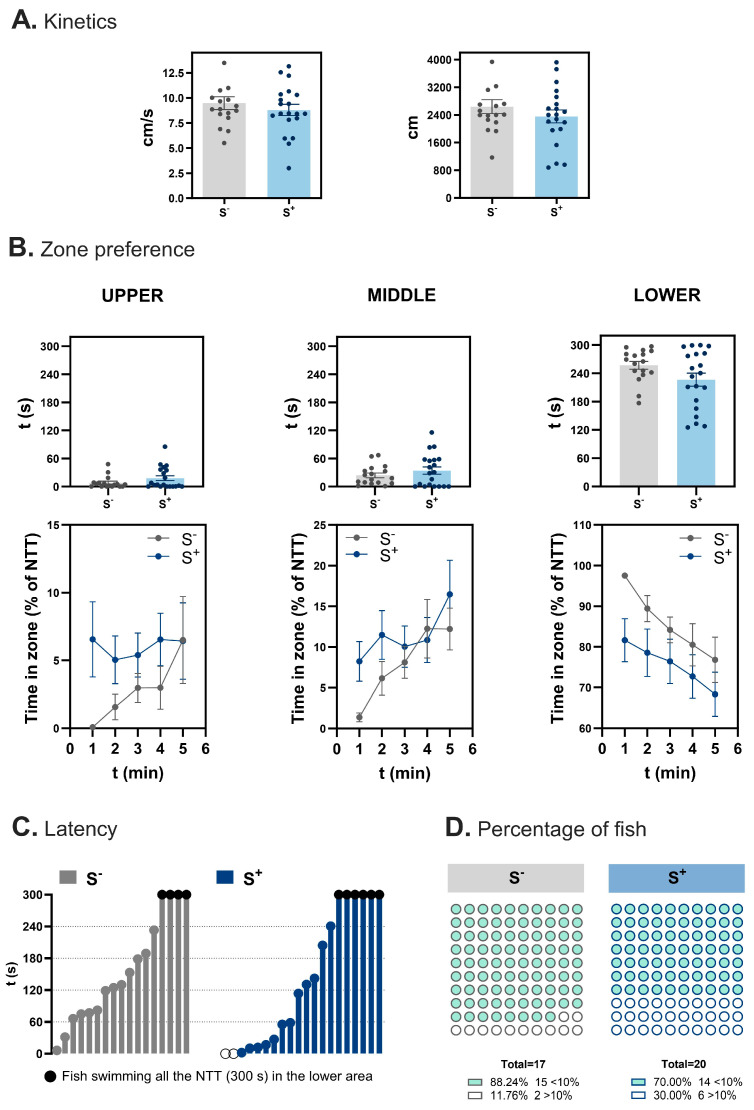
Behavioral studies in adult zebrafish by Novel Test Tank (NTT) Noldus Ethovision^®^ software, XT16 version (1 min acclimatization + 5 min evaluation). (**A**) Kinetic parameters: velocity (cm/s) and swum distance (cm). (**B**) Total time spent by fish in the upper, middle, and lower zones (s) and percentage of time (%) in each zone at different minutes. (**C**) Latency to the first entry in the upper zone. Note that individuals not reaching the upper zone are shown with black dots in the figure. (**D**) Percentage of fish (colored dots) spending less than 30 s (10% of the total NTT) in the upper zone during the behavior assay in each experimental group. S^−^: adult fish resulting from crossings involving control progenitors. S^+^: adult zebrafish from crossings involving control females and chronically stressed males. Data are presented as mean ± SEM.

We conducted a behavioral test based on the analysis of exploration behavior in a novel tank test (NTT) in larvae from both experimental groups (Figure 4B). We examined the obtained tracks (Figure 4C) individually in terms of the virtual grid (Figure 4D), focusing on the whole new area (all), the peripheral zone (outer), and the central zone of the explorable arena (inner). We found no statistically significant differences either in the global arena (*p* = 0.9316), outer (*p* = 0.6779), or inner (*p* = 0.9162) scoring comparison (Figure 4E). Thus, motile larvae from each experimental condition showed similar behavior patterns with a general scoring around 80% of the new exploration arena in both groups. In regard to adults, the 21-day stress protocol in the NTT induced in male progenitors did not trigger significant treatment effects in derived progenies (Figure A1). We found no difference between the control and CS fish (*p* > 0.0500) in kinetic parameters (velocity and swum distance). The fish mean velocity remained close to 8 cm/s and the reported distance swam was close to 2400 cm (Figure A1A). In addition, we detected no significant different swimming patterns depending on swimming zone preference, latency patterns, or the percentage of fish spending less than 30 s in the upper zone between the experimental groups (Figure A1B–D).

### 2.7. Cartilage Development

To further investigate the impact of stress on cranioencephalic development in zebrafish larvae, we conducted alcian blue staining which specifically targets cartilage, the primary structural tissue during this stage of development. We focused on three different parameters in this evaluation: Meckel’s–palatoquadrate (M–PQ) angle, ceratohyal cartilage length, and lower jaw length (Figure 4F). The staining process did not provide clear evidence of pronounced abnormalities in the cartilage structure, as depicted in Figure 4G. Upon qualitative examination, the malformed larvae showed no statistically significant differences between groups.

## 3. Discussion

Adverse situations induce a stress response in fish that begin with molecular and physiological changes to compensate the stressor and re-establish homeostasis [34]. The physiological response to stress is polymorphic between individuals in rate or magnitude; however, general characteristics in mode and action are shared among species [35]. The potential deleterious effects of CS on reproductive performance have been widely studied [36,37,38], yet knowledge is scarce regarding stress paternal transmission to unexposed progeny in fish and its mechanism.

We aimed to determine the potential role of miRNAs in the paternal inheritance of stress consequences in offspring. To achieve this, we performed a small RNA-seq study in the progenies derived from CS-exposed progenitors. We found a unique miR differentially expressed: miR-29a (Figure 2A–C), known for its multiple roles modulating cell proliferation, apoptosis, angiogenesis, oxidative stress, and fertility in mammals [39,40,41,42,43]. Considering this miR role in fertility, together with its potential function in progeny transmission, we decided to investigate whether its expression was altered in spermatozoa and testes from stressed progenitors (Figure 2C). Our results show no evidence of this; we obtained similar miR-29a expression patterns between the control and stressed males (Figure 2C), discarding its role as potential vehicle of phenotype inheritance.

To determine the potential consequences of this diminished miR-29a expression on mRNA regulation, we performed different in silico analyses in the zebrafish 7 dpf larvae derived from crossings involving chronically stressed males with control undisturbed females. Considering the multiple miR-29a targets, we performed different enrichment analyses using gProfiler to shed light on miR-29a deregulation consequences (Figure 2D–G). When we considered the possible molecular functions controlled by this miR, two main processes were noticed: protein binding and extracellular matrix structural constituent (Figure 2D). In terms of cellular components, predicted mRNAs controlled by miR-29a are mainly involved in cell membrane elements—such as membrane and cytoplasm—and extracellular matrix organization linked to collagen organization, confirmed by the Reactome pathway database (Figure 2E,F). More importantly, most of the biological processes affected were related to the (i) developmental process and morphogenesis, (ii) nervous system development, and (iii) extracellular matrix organization (Figure 2G). For this reason, we performed a qPCR analysis in a first molecular approach to validate some genes targeted by miR-29a in these affected biological processes, molecular functions, or cellular components.

The results from these analyses indicate an upregulation of some targets related to developmental processes in offspring derived from stressed males. Target genes were overexpressed in larvae from disturbed parents (Figure 3A), especially those involved in the formation of some structures—such as eye (*emilin3)* and axon and dendrites (*map1b*)—and different markers involved in cell proliferation and DNA replication (*e2f7*, *tpx2,* and *atad5*) [44,45,46]. We observed the same pattern in those transcripts linked to nervous system development and neuron projection morphogenesis (*col8a1b* and *map1b*) [47,48,49] in our indirectly stressed progenies (Figure 3A). Moreover, the same higher expression was observed in 7 dpf larvae from disturbed males in different components of extracellular matrix—*col8a1a*, *nefm*, and *col91b*—compared with that in the control progenies. These observed overexpression patterns in the offspring from disturbed males validated the in silico analyses for target prediction.

In a final approach, we explored whether these molecular alterations in miR-29a targets observed in progenies from stressed males could be translated into phenotypic abnormalities related to these biological processes or cellular components. Cell division, differentiation, proliferation, and morphogenesis are coordinated events during zebrafish embryonic development and appear frequently in disarray in different pathologies such as cancer. The role of morphogenesis in zebrafish embryo survival and hatching rates has been widely studied and reviewed [50,51,52]. We found a diminished survival in larvae from stressed parents accompanied by lower values of hatching rates (Figure 3B,C), confirming our in silico data and molecular analyses. Our results agree well with recent research indicating that certain types of direct stress—specifically cold stress—can affect this process of morphogenesis, triggering decreased survival ratios in zebrafish larvae [52]. In the present work, we determine the same effects on the development, survival, and hatching rates under indirect stress conditions in zebrafish.

Regarding the nervous system altered pathways we found, first, we evaluated the status of miR-29a in the brain of stressed males compared with that in the brain of undisturbed ones (Figure 4A). We did not find significant differences in its expression between experimental groups (Figure 4A). However, we obtained an altered expression pattern of some miR-29a targets related to nervous system development in larvae from stressed males (Figure 3A). These molecular findings could be correlated to anxiety behavior observed in directly exposed progenitors to the stress protocol assessed by an NTT and published in a previous study [13]. Thus, we analyzed the swimming pattern of 7 dpf larvae (Figure 4) and adult (Figure A1) zebrafish derived from stressed parents to discard neural disorders associated with the expression abnormalities found [53,54,55]. This type of behavioral test represents the simplest assay to quantify zebrafish locomotor activity stimulated by changes in their central nervous system [53,55]. We observed neither a substantial impact on larvae swimming patterns (Figure 4A–D), nor evidence of behavior disturbances in zebrafish adults (Figure A1).

Finally, we investigated whether the enriched expression of some miR-29a targets, described as components of extracellular matrix (*col8a1a, col9a1b,* and *nefm*; Figure 3A), had any phenotypic consequences in zebrafish 7 dpf larvae derived from crossings involving chronically stressed males with control undisturbed females. Previous investigations have described the role of collagen 9 in extracellular matrix synthesis, determining the shape of the jaw in zebrafish, regulating cartilage morphogenesis and mineralization [56,57,58]. A downregulation of miRNA-29a in fish bone cells can decrease the expression of BMP2, oseteocalcin, and osteopontin genes, leading to diminished cell differentiation and extracellular matrix mineralization [58]. We performed a cartilage staining to explore whether alterations in these molecular pathways were translated at tissue level in jaw morphology in our unexposed larvae from stressed males. We cannot confirm any modification pattern in jaw shape in this group in terms of ceratohyal cartilage length, palatoquadrate angle, or lower jaw length (Figure 4F,G). These measures are considered high-throughput standard parameters to assess craniofacial malformations in zebrafish [59].

Our findings indicate (i) a vertical transmission of chronic stress via sperm to the unexposed larvae; (ii) novel consequences of paternally inherited chronic stress at molecular level—miR and mRNA—that suggest development, morphogenesis, and neurogenesis as potentially disrupted processes; and (iii) final phenotypic alterations entailing reduced survival and hatching rates. The deregulation of miR-29a could affect relevant biological processes of great importance in the field of aquaculture since the alterations in early development can negatively affect sector sustainability at an economic level. Moreover, the present work shows that the existence of negative stimuli in aquaculture can predispose not only the exposed fish but also their offspring to compromised growth, and eventually promote higher mortality rates, decreasing hatchery production efficiency.

Further investigations should be conducted to validate the putative target genes described in the present study. Some of the approaches described herein could shed light on an important challenge of miRNA biology, contributing to identifying the rules of miRNA target recognition. In addition, the functional validation of these targets would strengthen our results, establishing their direct involvement in the observed phenotypic changes.

## 4. Materials and Methods

### 4.1. Animal Maintenance and Experimental Design

For the present work, all experiments were performed in adult (12 months) zebrafish (*Danio rerio*; AB wildtype strain). Fish maintenance was performed under standard laboratory conditions (pH 7.0–7.5; salinity 400–500 μS; temperature 26–29 °C [60]) in a multitank recirculating water system (Aquatic Habitats, Speonk, NY, USA). Animals were kept on a 14:10 h light/dark photoperiod cycle and were fed twice a day with commercial pelleted feed, Zebrafeed (Sparos, Olhão, Portugal).

We conducted the experiment in three stages (Figure 1). The aim of Stage 1 was to discover altered noncoding RNAs in the 7 dpf progenies derived from chronically stressed males crossed with control females. Stage 2 aimed to evaluate the resulting noncoding RNA profile in the following paternal biological samples: sperm, brain, and testicles. Stage 3 aimed to explore the physiological changes linked to the altered molecules found between the control- and stress-derived larvae.

We randomly divided 30 adult male zebrafish into two groups: control (S^−^) and chronically stressed (S^+^), with 15 males per group. Both groups were placed into separate tanks for a habituation period (1 month). Afterwards, fish in the stressed group (S^+^) were exposed to a CS protocol for 21 days to cover several stages of a wave of spermatogenesis in this species [61]. CS was defined as a predictable handling source of stress twice a day and a main body of unpredictable chronic stress (UCS). We used the UCS protocol previously published by our group [13], an adaptation of a previously described protocol for this model species [62] based on three of its iterations. The stressors combination represents common situations under captive conditions in the aquaculture industry or in the natural environment. We placed S^+^ fish in an induction tank—expected handling source of stress—twice a day (9 am and 14 pm) and subjected them to an acute stressor from the following list: cool water (23 °C; 30 min), warm water (33 °C; 30 min), dorsal body exposure to air (low water level; 2 min), persecution (net in the exposure tank; 8 min), crowding (250 mL beaker; 50 min), three consecutive tank changes (30 min each), and exposure to predator *Archocentrus nigrofasciatus* (video recording close to the induction tank; 50 min). Simultaneously, the control group (S^−^) remained undisturbed in their housing tanks; anthropogenic-derived stimuli were therefore decreased by the reduction in handling and human occurrence near the tanks.

Once the CS protocol finished, we crossed four males from each group (S^+^ and S^−^) from each replicate with control undisturbed females (1♂:1♀ ratio) to obtain progeny following standard breeding protocols [60]; 4–5 males were euthanized and their brains and testes were dissected and stored for further molecular analysis, while the rest of the batch were squeezed for sperm collection following standard protocols. We considered the F1 progeny resulting from each crossing as a biological replicate (*n* = 4–5). Larvae were incubated until 7 dpf at 28 ± 1 °C. We studied two parameters during this timeframe to assess progeny development: embryo hatching at 72 dpf and daily survival rate (0–7 dpf). We repeated the experiment twice, obtaining 4–5 biological replicates per batch. The first experimental replicate was used for small RNA-seq analysis and tissue storage for following noncoding RNA profile evaluation. The second experimental replicate was used for physiological larvae evaluation (behavior and cranioencephalic development analysis). We collected data regarding larvae survival and hatching from both experimental replicates.

### 4.2. RNA Isolation

We performed total RNA isolation equally for all biological samples—7 dpf larvae, brain, testicles, and sperm—using the miRNeasy tissue kit (Qiagen, Madrid, Spain). The protocol included a first dissociation step with Qiazol Lysis Reagent (Qiagen). The 7 dpf larvae samples were composed of a pool of 50 individuals, and sperm samples were composed of a pool of 4 ejaculates. We used the CNAG-CRG services to examine the quality of the 7 dpf larvae samples using Experion (Experion™ Automated Electrophoresis System, BioRad, Madrid, Spain). Only samples meeting the requirements (3 µg of RNA; RNA integrity number (RIN) > 8) were used. An aliquot of each RNA sample was used for the small RNA-seq experiment and another was stored for qPCR experiments. RNA samples from brain, gonads, and sperm for qPCR experiments were isolated using the same protocol and commercial kit. We measured the quality and purity of these samples with a NanoDrop™ One/OneC spectrophotometer (Thermo Fisher Scientific™, Madrid, Spain), ensuring that the isolated RNA showed high purity (A260/280 > 2.0), and examined them in an agarose gel.

### 4.3. Small RNA-Seq Library Preparation and Sequencing

Stranded mRNA library preparation and sequencing were performed in the CNAG-CRG platform. We determined the quantity and quality of the total RNA sample using the Qubit RNA BR Assay kit (Thermo Fisher Scientific, Madrid, Spain) and RNA 6000 Nano Bioanalyzer 2100 Assay (Agilent, Santa Clara, CA, USA). The RNA-seq libraries were prepared with KAPA RNA HyperPrep Kit with RiboErase (Roche, Madrid, Spain) following the manufacturer’s recommendations, using Illumina platform compatible adaptors with unique dual indexes and unique molecular identifiers (Integrated DNA Technologies, IA, USA). The final library was validated on an Agilent 2100 Bioanalyzer with the DNA 7500 assay. The libraries were sequenced on NovaSeq 6000 (Illumina, Berlin, Germany) following the manufacturer’s protocol for dual indexing. Image analysis, base calling, and quality scoring of the run were processed using the manufacturer’s software Real Time Analysis (RTA v3.4.4) and followed by generation of FASTQ sequence files.

### 4.4. Small RNA-Seq Data Processing and Data Analysis

Small RNA-seq reads were trimmed using Trim-Galore 0.6.6, with a minimum length of 16 bp and stringency of 10 to remove adaptors. These trimmed reads were mapped against zebrafish reference genome (GRCz11) using STAR aligner version 2.7.8a with ENCODE parameters for small RNA. We quantified the annotated genes using ensemble release 104 annotation, with software RSEM version 1.3.0 and default parameters. Differential expression analysis between four stressed and four control samples was performed with limma using the voom transformation of the counts. Genes with log2FC > |0.58| and FDR < 5% were considered differentially expressed genes. Gene set enrichment analysis was performed using a preranked list of genes sorted by the limma moderated t statistic and the fgsea R package. The multidimensional scaling plot was conducted with the limma R Package.

### 4.5. Retrotranscription

miR retrotranscription was conducted in 7 dpf larvae brain, testicles, and sperm. A specific Taqman Small RNA probe (5×) was employed for each miR (dre-miR-29a and dre-miR-92a), in accordance with the manufacturer’s instructions (initial amount of total RNA: 10 ng). We used the High-Capacity cDNA Reverse Transcription Kit (Applied Biosystems, Madrid, Spain) for 7 dpf larvae mRNA retrotranscription (initial amount of total RNA: 1 μg), following the kit guidelines. Both products were stored at −20 °C until further use.

### 4.6. Target Prediction of miR-29a and Functional Enrichment Analysis

We specifically directed the target prediction search towards miR-29a based on RNA-seq results. TargetScanFish software (6.2. version, Whitehead Institute, Cambridge, UK) was employed to compile the reported mRNA targets of miR-29a. The resulting list of targets was then used as a query in the g:Profiler analysis tool. The functional enrichment analysis was performed using g:Profiler (version e109_eg56_p17_1d3191d) with g:SCS multiple testing correction method applying a significance threshold of 0.05 [63]. The analysis was configured to explore molecular function, cellular component, and gene ontology (GO) biological processes using the REACTOME database for GO analyses.

### 4.7. Primers Design

We selected nine miR-29a-targeted genes for gene expression studies: collagen, type VIII, alpha 1a (*col8a1a*); collagen, type IX, alpha 1b (*col9a1b*); TPX2 microtubule nucleation factor (*tpx2*); E2F transcription factor 7 (*e2f7*); microtubule-associated protein 1B (*map1b*); neurofilament medium chain b (*nefmb*); ATPase family AAA domain containing 5a (*atad5a*); *si:ch211-203k16.3*; and elastin microfibril interface 3a (*emilin3a)*. Specific primer pairs were designed using the NCBI Primerblast tool. GenBank references, primers sequences, amplicon length, and melting temperature are presented in Table 1.

### 4.8. qPCR Analysis

We performed qPCR experiments in a StepOnePlus Real-Time PCR System (Applied Biosystems, Madrid, Spain) under standard thermal conditions. Technical triplicates were conducted for each sample (*n* = 4 biological replicates).

#### 4.8.1. qPCR Analysis for miRNAs

Reactions were prepared according to the protocol for Taqman Universal PCR master mix II (Applied Biosystems).

#### 4.8.2. qPCR Analysis for mRNAs

The composition of each reaction (20 μL) was 10 μL of SYBR Green PCR Master Mix (Applied Biosystems, Madrid, Spain), 1 μL of each 10 μM forward and reverse primer (Table 1), 2 μL of cDNA sample, and 6 μL of Molecular Biology degree water up to 20 μL. We performed a melting curve analysis to determine the specificity of qPCR reactions. Gene expression was calculated relative to actb2 as housekeeping gene following the Pfaffl’s mathematical method [64].

### 4.9. Progeny Evaluation

From 1 to 7 dpf, any deceased embryos or larvae were removed from the culture dishes. We assessed the hatching rate—percentage of larvae that successfully hatched in relation to the total number of live animals in the batch—at 72 hpf. The evaluation of hatching and survival rates was conducted using a Nikon^®^ SMZ1500 stereomicroscope (Nikon, Tokyo, Japan).

### 4.10. Larvae and Adult Behavior Analyses

We assessed the swimming activity of the larvae at 7 dpf. A previously described NTT for zebrafish larvae [65] was used with a 5 min evaluation time. A total of 24 larvae were randomly selected for evaluation from 3 biological replicates.

Each larva was individually placed in a novel Petri dish and allowed to acclimate to the new environment for 1 min. Subsequently, the behavior of each larva was recorded for 5 min. The swimming tracks of the larvae were then monitored and analyzed using Tracker software 6.0.8 version (physlets.org/tracker/). To compare fish exploration, each resulting swimming track was processed using a virtual grid pattern comprising 16 zones, categorized as 8 outer and 8 inner zones. Mean values resulting from the 24 larvae from each biological replicate were compared between the two experimental groups.

When larvae reached adult stage (12 months), we performed an NTT to analyze the effects of stress induction in zebrafish parents on their progenies in terms of anxiety levels. Fish were individually placed at the bottom of a trapezoidal transparent tank (width: 11 cm; height: 17.5 cm; length at top: 28 cm) containing 3.5 L of aquarium water. We changed the water after three trials to diminish variations in temperature (~28 °C) and avoid presence of stress hormones from already trialed individuals. Zebrafish behavioral activity was recorded (1920 × 1080 px) for 6 min. The resulting videos were processed with Noldus Ethovision^®^ XT16 (Noldus Information Technologies, Inc., Leesburg, VA, USA) tracking software. The first minute was considered an acclimatization period and was not considered in the analysis. The tank area was split into three virtual zones (upper, middle, and lower) to offer an exhaustive evaluation of vertical swimming activity. Velocity of fish, distance swum, time spent in the different zones (percentage of NTT), and latency were the parameters registered.

### 4.11. Alcian Blue Cartilage Staining

To analyze the development of cartilage in the larvae, whole mount alcian blue staining was conducted on 7 dpf larvae [65]. Specimens from both experimental groups were fixed in 4% phosphate buffered paraformaldehyde at 4 °C overnight. Following fixation, the samples were stored in 70% ethanol at 4 °C. Stained larvae (*n* = 3–5 per biological replicate; 7–9 biological replicates) were positioned ventrally under a SMZ1500 stereomicroscope (Nikon, Tokyo, Japan) and photographed using a Nikon DS-Fi3 camera (Nikon Instruments Inc., Tokyo, Japan). Resulting images were processed using ImageJ software 1.46 version [66]. Measurements of head length, ceratohyal cartilage length, lower jaw length, and M–PQ angle were obtained for each larva.

### 4.12. Statistics

We employed the GraphPad Prism 9.0.0 package (GraphPad Software, Inc, Boston, MA, USA.) for result representation and statistical analyses. All studied variables were analyzed to ensure a normal distribution using a Shapiro–Wilk normality test. When the experimental groups (S^−^ and S^+^) passed the normality test, significant differences between them were detected using Student’s *t*-test. For nonparametric data, we performed a Mann–Whitney test. Survival curve comparison was explored using a log-rank Mantel–Cox test. All data are presented as mean ± SEM (* *p* < 0.0500; ** *p* < 0.0100; *** *p* < 0.0010; ns: not significant changes).

## Figures and Tables

**Table 1:** Primer pairs used in this study.

Gene	Accession Number	Oligo	Sequence (5′ to 3′)	Amplicon (bp)
*atad5*	XM_003198109.4	F	GGTGTTTGGCGCCATTTG	98
		R	TCTGAAGTGCGCTTGAACCT	
*col8a1a*	NM_001142374.1	F	AAGGTGTGTGTTTTCGGGGT	185
		R	TTGCTGAGGATGCGACTTGT	
*si:ch211-203k16.3*	XM_021468697.1	F	GCAGGCGTTTTGGAAGGTTT	206
		R	ACCCCATCTTAAGAAACATGTGC	
*col9a1b*	NM_213264.2	F	GATCTGGGACTCCTGGTCCT	165
		R	TGGGGCCTATGAGACCATCA	
*e2f7*	NM_001045147.2	F	ACAGGGACTTTTACAGGCCG	282
		R	TACAAACGCCGCACCTTAGT	
*emilin3a*	XM_021474148.1	F	ACGAGTGGAAAGTGCAGAGG	132
		R	GCCCTCAATCTTTCAGCCCT	
*map1b*	NM_001128201.2	F	AACCACACCACCAAAGCTCA	188
		R	CCATAGGCGTTGGGACACTT	
*nefmb*	NM_001123280.1	F	CGGTGCGCAATAACGAGAAG	83
		R	CTCGAGGAAGCGAACCTTGT	
*tpx2*	NM_001327745.1	F	AGATCAGTCATGCAGGTGCT	72
		R	AGCCAACTCTCCTCTAGGCA	

## Data Availability

Small RNA-seq data can be found in: https://www.ncbi.nlm.nih.gov/geo/query/acc.cgi?acc=GSE240954 (accessed on 17 August 2023).
